# Periods of Incubation of the Communicable Diseases

**Published:** 1882-01

**Authors:** 


### PERIODS OF INCUBATION OF THE COMMUNICABLE DISEASES.

Dr. B. W. Richardson gives a list of twenty-five communicable diseases which have a period of incubation. He adds a list of eleven diseases concerning which it cannot be said certainly that they have a period of incubation. These latter are: catarrh, puerperal fever, pyæmia, hospital gangrene, sloughing phagedæna, remittent fever, intermittent fever, choleraic diarrhœa, cerebro-spinal fever, carbuncle.

The diseases attended with stages of incubation are conveniently divided into five groups:—

*Shortest.*—Incubation one to four days; Malignant cholera, malignant pustule, plague, catarrh, dissection wound disease.

*Short.*—Incubation two to six days: Scarlet fever, rosalia idiopathica, diphtheria, dengue, erysipelas, yellow fever, pyæmia, influenza, pertussis, glanders, farcy, crease, croup, puerperal fever.

*Medium.*—Incubation five to eight days: Relapsing fever, gonorrhœa, vaccinia, inoculated variola.

*Long.*—Incubation ten to fifteen days: smallpox, varicella, measles, rœtheln, typhus, typhoid, mumps, malarial fever.

*Longest.*—Incubation forty days or more: Syphilis, hydrophobia. —*Med. Times.*

